# Whole blood DNA methylation profiles of Alzheimer’s disease differ across ancestrally diverse populations

**DOI:** 10.1002/alz.092713

**Published:** 2025-01-03

**Authors:** Tianjie Gu, Lissette Gomez, Makaela Mews, Patrice L. Whitehead, Kara L. Hamilton‐Nelson, Jose Javier Sanchez, Larry D. Adams, Pedro R. Mena, Takiyah D. Starks, Concepcion Silva‐Vergara, Maryenela Z. Illanes‐Manrique, Michael L. Cuccaro, Jeffery M. Vance, Mario Cornejo‐Olivas, Goldie S. Byrd, Briseida E. Feliciano‐Astacio, Jonathan L. Haines, Gary Beecham, Margaret A Pericak‐Vance, William S. Bush, Anthony J. Griswold

**Affiliations:** ^1^ John P. Hussman Institute for Human Genomics, University of Miami Miller School of Medicine, Miami, FL, USA, Miami, FL USA; ^2^ John P. Hussman Institute for Human Genomics, University of Miami Miller School of Medicine, Miami, FL USA; ^3^ System Biology & Bioinformatics; Department of Nutrition; Case Western Reserve University, Cleveland, OH USA; ^4^ Maya Angelou Center for Health Equity, Wake Forest University School of Medicine, Winston‐Salem, NC USA; ^5^ Universidad Central del Caribe, Bayamón, PR USA; ^6^ Neurogenetics Research Center, Instituto Nacional de Ciencias Neurológicas, Lima Peru; ^7^ Dr. John T. Macdonald Foundation Department of Human Genetics, University of Miami Miller School of Medicine, Miami, FL USA; ^8^ Neurogenetics Working Group, Universidad Científica del Sur, Lima Peru; ^9^ Department of Population and Quantitative Health Sciences, Institute for Computational Biology, Case Western Reserve University, Cleveland, OH USA; ^10^ 1501 NW 10th Avenue, Miami, FL USA

## Abstract

**Background:**

Prior studies have shown differences in the genetic etiology and clinical presentation of Alzheimer’s Disease across populations. For example, for multiple genetic loci associated with AD, effect sizes can vary drastically between individuals of different ancestral backgrounds. Few investigations into differences in epigenetic features like DNA methylation have been conducted in AD, particularly in diverse individuals. These studies are critical to identify and further characterize mechanisms of disease allowing for development of therapeutic interventions.

**Method:**

As part of an ongoing study of the genetics and epigenetics of AD in diverse populations, we performed a methylome analysis of 626 individuals. DNA from whole blood was analyzed using the Illumina MethylationEPICv2.0. The cohort consisted of both AD and cognitively unimpaired (CU) individuals of European (68 AD, 67 CU), African (98 AD, 106 CU), or Hispanic (Puerto Rican (85 AD, 76 CU); Peruvian (41 AD, 41 CU); Cuban (22 AD, 22 CU)) backgrounds. We analyzed data using the SeSAMe R package for quality control and statistical analysis. We performed differential methylation analysis between AD and CU in the overall dataset and within each ancestral population using linear models with covariates sex, age of exam, batch effect, global ancestry and estimated immune cell type proportions.

**Result:**

878,853 CpG sites were tested for differences between AD status. In a preliminary analysis of these data, we identified 563 CpG sites with nominally significant differences (p‐value ≤ 0.001) between AD and CU. Within each ancestral group, the number of differentially methylated sites differed: European – 442 sites, African – 217 sites, Hispanic – 475. Notably, however, the markers within the ancestral group did not overlap, implying that the AD disease process may be quite different across populations.

**Conclusion:**

While these results are preliminary, and expansion of the dataset may reveal convergence of methylation patterns across ancestral populations, our analyses suggest the possibility of ancestry specific whole blood DNA methylation patterns as signatures of AD pathogenesis. Ultimately, combining these methylation profiles with existing genomic and transcriptomic data may reveal distinct genes but similar underlying pathological processes contributing to AD across individuals of diverse ancestries.

